# Amide-bridged conjugated organic polymers: efficient metal-free catalysts for visible-light-driven CO_2_ reduction with H_2_O to CO†

**DOI:** 10.1039/d1sc02499j

**Published:** 2021-07-13

**Authors:** Fan Wen, Fengtao Zhang, Zhen Wang, Xiaoxiao Yu, Guipeng Ji, Dongyang Li, Shengrui Tong, Yingbin Wang, Buxing Han, Zhimin Liu

**Affiliations:** Beijing National Laboratory for Molecular Sciences, Key Laboratory of Colloid and Interface and Thermodynamics, CAS, Research/Education Center for Excellence in Molecular Sciences, Institute of Chemistry, Chinese Academy of Sciences 100190 Beijing P. R. China liuzm@iccas.ac.cn; School of Chemistry, University of Chinese Academy of Sciences Beijing 100049 P. R. China; School of Science, China University of Geosciences Beijing 100083 P. R. China

## Abstract

The visible-light-driven photoreduction of CO_2_ to value-added chemicals over metal-free photocatalysts without sacrificial reagents is very interesting, but challenging. Herein, we present amide-bridged conjugated organic polymers (amide-COPs) prepared *via* self-condensation of amino nitriles in combination with hydrolysis, for the photoreduction of CO_2_ with H_2_O without any photosensitizers or sacrificial reagents under visible light irradiation. These catalysts can afford CO as the sole carbonaceous product without H_2_ generation. Especially, amide-DAMN derived from diaminomaleonitrile exhibited the highest activity for the photoreduction of CO_2_ to CO with a generation rate of 20.6 μmol g^−1^ h^−1^. Experiments and DFT calculations confirmed cyano/amide groups as active sites for CO_2_ reduction and second amine groups for H_2_O oxidation, and suggested that superior selectivity towards CO may be attributed to the adjacent redox sites. This work presents a new insight into designing photocatalysts for artificial photosynthesis.

## Abstract 2

Amino nitrile-derived conjugated organic polymers can realize the photoreduction of CO_2_ with water to CO without H_2_ generation efficiently.

## Graphical abstract

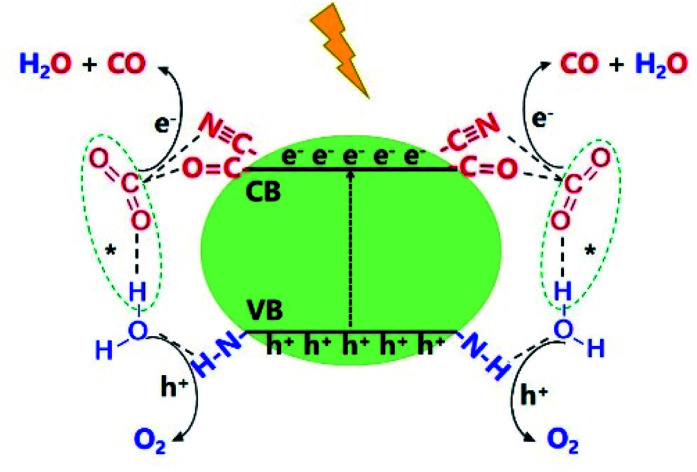

## Introduction

The massive consumption of fossil fuels has caused excessive CO_2_ emissions, and the CO_2_ issue has attracted worldwide attention.^1^ The photocatalytic reduction of CO_2_ with H_2_O to value-added chemicals is a promising and ideal way to realize CO_2_ transformation and has received much interest.^2–4^ To date, various kinds of photocatalysts including metal oxide/sulfide semiconductors,^5,6^ perovskite materials,^7^ metal–organic frameworks,^8,9^ and conjugated organic polymers (COPs)^10,11^ have been developed for the photocatalytic reduction of CO_2_. COPs as a class of metal-free materials with wide application have attracted continuous interest because of their high designability.^12,13^ Especially, COPs with CO_2_-philic functional groups (*e.g.* triazine, azo, amine and cyano groups) have been reported to show good performances in CO_2_ capture and transformation,^14,15^ and some heteroatom (*e.g.*, N, P and S) doped COPs can realize CO_2_ photoreduction under visible light irradiation.^16,17^ For example, hexachlorocyclo-triphosphazene-derived COPs showed high CO_2_ absorption capacity and could photocatalyze CO_2_ reduction to CH_4_ under visible light irradiation in the presence of triethanolamine.^18^ So far, only a few photocatalysts can achieve the reduction of CO_2_ with H_2_O,^10,19,20^ and sacrificial reagents are required in most cases. Therefore, it is very demanding to efficiently couple the photoreduction of CO_2_ with the photooxidation of H_2_O in one photocatalytic system, especially under visible light irradiation. The natural photosynthesis of CO_2_ with H_2_O produces biomass with O_2_ release and no H_2_ generation. However, in the artificial photocatalytic process of CO_2_ with H_2_O the generation of side reaction products and H_2_ is hardly avoided. If the CO_2_ reduction could couple with the H_2_O oxidation perfectly, the H_2_ generation may be suppressed. To achieve this goal, a photocatalyst is the key, but such a photocatalyst is seldom reported.^11^

**Scheme 1:**
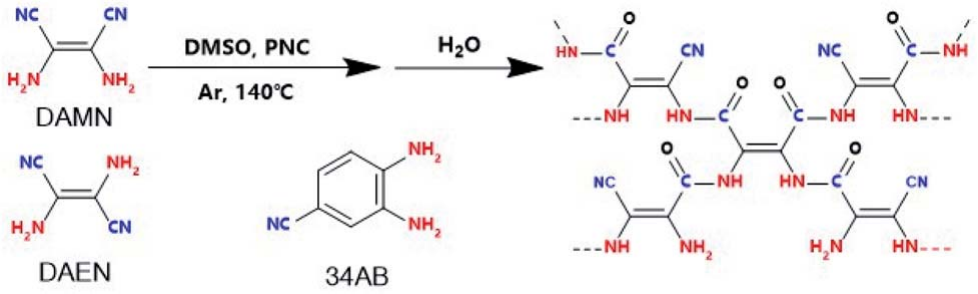
Synthetic procedure for amide-COP production.

Herein, we report amide-bridged COPs (amide-COPs, including amide-DAMN, amide-DAEN and amide-34AB) with –C–NH–(C=O)–C—as the structural unit and rich in the –CN group, for the photoreduction of CO_2_ with H_2_O, which were prepared *via* self-condensation of amino nitriles (*i.e.*, diaminomaleonitrile, DAMN; 2,3-diaminobut-2-ene-1,4-dinitrile, DAEN; 3,4-diaminobenzonitrile, 34AB) in combination with subsequent hydrolysis,^21,22^ as illustrated in Scheme 1. These COPs could absorb visible light efficiently and exhibit high CO_2_ adsorption capacities. Importantly, they could realize the photoreduction of CO_2_ with H_2_O without any photosensitizer or sacrificial reagent under visible light irradiation, affording CO as the sole carbonaceous product without H_2_ generation. Especially, amide-DAMN with a suitable energy band structure (*E*_g_ = 2.19 eV, CB = −0.75 eV, and VB = 1.44 eV) displayed the highest activity for the photoreduction of CO_2_, affording CO with a production rate of 20.6 μmol g^−1^ h^−1^, much better than most reported metal-free catalysts. Density functional theory (DFT) calculations indicate that the adjacent redox sites of this kind of photocatalyst make the CO_2_ photoreduction couple well with H_2_O photooxidation, resulting in no H_2_ generation.

## Results and discussion

**Fig. 1:**
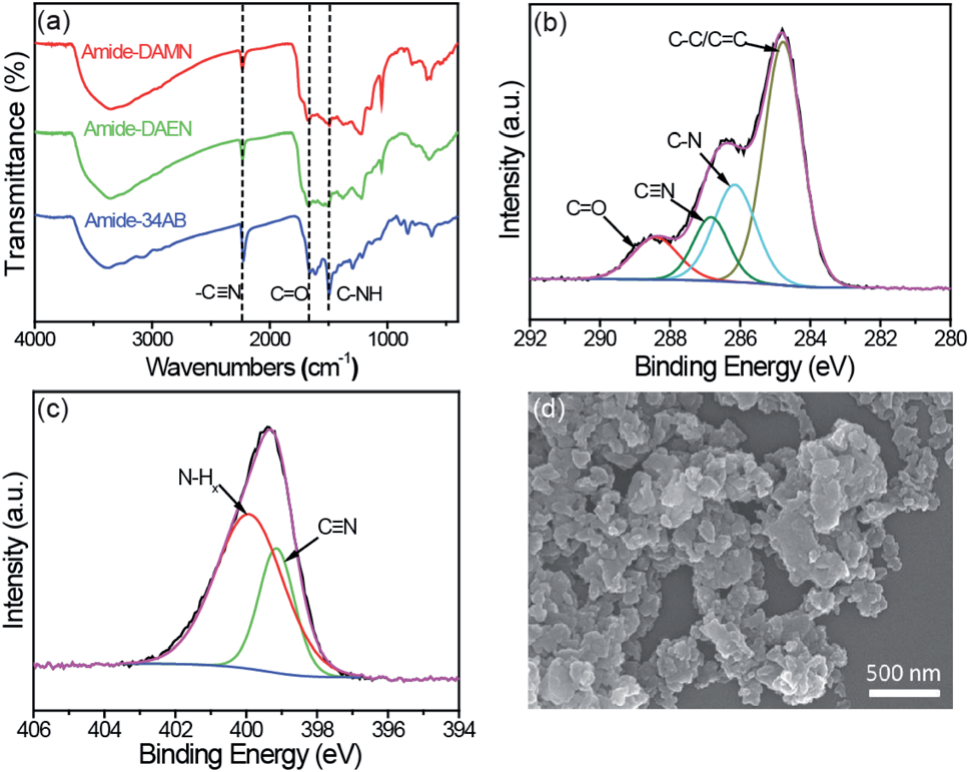
(a) FT-IR spectra of amide-COPs. (b) High resolution XPS C 1s spectrum of amide-DAMN. (c) High resolution XPS N 1s spectrum of amide-DAMN. (d) SEM image of amide-DAMN.

The formation of amide-COPs was confirmed by Fourier-transform infrared (FT-IR), cross-polarization magic-angle spinning (CP/MAS) ^13^C NMR and X-ray photoelectron spectroscopy (XPS) analyses. In the FT-IR spectra (Fig. 1a and S1†), the cyano signal at around 2228 cm^−1^ of each polymer was significantly weakened as compared with that of the corresponding monomer, and new peaks appeared at 1672 and 1501 cm^−1^ corresponding to the stretching vibration of the C=O bond and the deformation vibration of N–H in the samples.^23,24^ In CP/MAS ^13^C NMR spectra (Fig. S2†), the sp^2^ hybridized vinyl carbon and aromatic carbon species in the benzene rings are located in the range of 105 to 158 ppm. The well-defined signals at 160–165 ppm and 116–119 ppm can be attributed to the carbon in the amide groups and cyano groups, respectively. The signals of the aliphatic area may be due to the residual DMSO in the material and the partially added sp^2^ hybridized carbon.^9,25^ In the C 1s XPS spectrum of amide-DAMN (Fig. 1b), four peaks with the binding energies (BEs) at 288.5, 286.8, 286.2, and 284.7 eV are deconvoluted, ascribing to the C=O, C≡N, C–N and C–C/C=C bonds, respectively.^26^ The deconvolution of the N 1s XPS spectrum (Fig. 1c) exhibits two peaks attributed to amino groups (N–H_*x*_, *x* = 1, 2) at 400.0 eV and to the C≡N group at 399.1 eV.^27,28^ Correspondingly, amide-DAEN and amide-34AB showed similar XPS spectra (Fig. S3†).

The powder X-ray diffraction (XRD) analysis revealed that amide-COPs were amorphous (Fig. S4†), and field-emission scanning electron microscopy (SEM) and transmission electron microscopy (TEM) (Fig. 1d and Fig. S5†) observations showed that the polymers were an assembly of nanoparticles with irregular shapes. Their N_2_ adsorption–desorption isotherms represent typical type IV isotherms with definite type H3 hysteresis loops (0.6 < *P*/*P*_0_ < 1) (Fig. S6†), indicating the coexistence of mesopores and macropores in the polymeric matrices, consistent with Barrett–Joyner–Halenda (BJH) pore size distribution. The Brunauer–Emmett–Teller (BET) surface areas of amide-DAMN, amide-DAEN and amide-34AB were determined to be 47, 60 and 31 m^2^ g^−1^, respectively (Fig. S7†). The CO_2_ adsorption capacities of amide-DAMN, amide-DAEN and amide-34AB reached 23.6, 14.5 and 32.7 mg g^−1^ at 1 bar at 273 K, respectively (Fig. S8†). The porous structures and high CO_2_ adsorption capacities of amide-COPs may be favorable to the adsorption and activation of H_2_O and CO_2_ molecules.

**Fig. 2:**
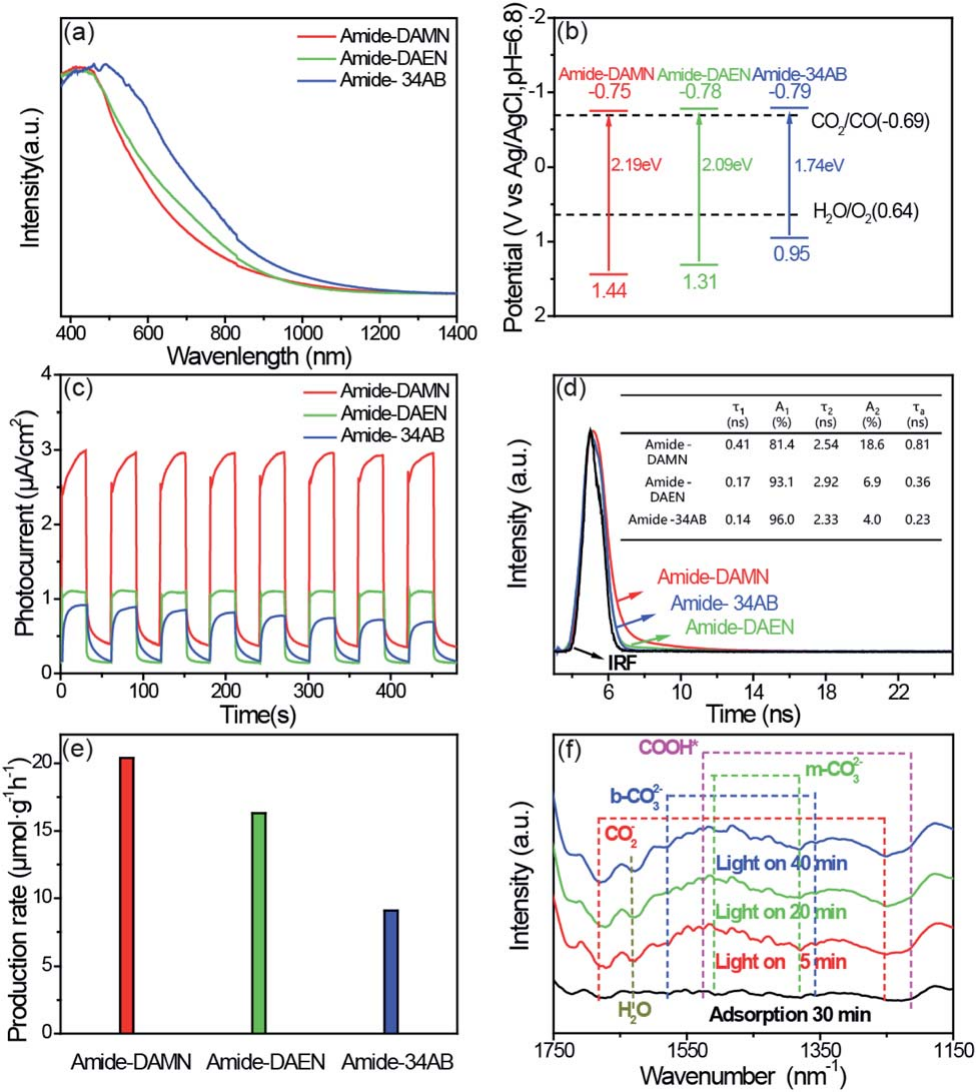
(a) UV/Vis light absorption spectra of amide-COPs. (b) Schematic band structure of amide-COPs. (c) Transient photocurrent responses of amide-COPs under visible light irradiation (*λ* > 420 nm). (d) Time-resolved PL spectra of amide-COPs. (e) Average product evolution rate over amide-COPs under visible light irradiation (*λ* > 420 nm). (f) *In situ* FT-IR spectra of amide-DAMN.

The photophysical properties of amide-COPs were measured by UV-vis diffuse reflectance spectroscopy (DRS). As illustrated in Fig. 2a, amide-COPs have strong capability to absorb visible light. Based on the Tauc plots, the optical band gaps of amide-COPs were estimated to be 2.19, 2.09 and 1.74 eV for amide-DAMN, amide-DAEN and amide-34AB, respectively (Fig. S9†). Applying Mott–Schottky analysis, the conduction band (CB) positions were determined to be located at −0.75, −0.78 and −0.79 eV (*vs.* Ag/AgCl, pH = 6.8) for amide-DAMN, amide-DAEN and amide-34AB, respectively (Fig. S10†), and the positive slopes of the plots demonstrated their typical characters of n-type semiconductors.^29^ Accordingly, the valence band (VB) positions of amide-DAMN, amide-DAEN and amide-34AB were calculated to be at 1.44, 1.31 and 0.95 eV (*vs.* Ag/AgCl, pH = 6.8), respectively. From their band gap structures (Fig. 2b), it seems that all amide-COP samples may be capable of powering the reduction of CO_2_ to CO (−0.69 eV *vs.* Ag/AgCl, pH = 6.8) and oxidation of H_2_O to O_2_ (0.64 eV *vs.* Ag/AgCl, pH = 6.8). This implies that amide-COPs may have the capability to catalyze the photoreduction of CO_2_ with H_2_O.

The separation efficiency of photogenerated electrons and holes and their migration rate are related to the activity of the photocatalyst. Electrochemical characterization including transient photo-current, electrochemical impedance spectroscopy (EIS) and steady-state and time-resolved photoluminescence (PL and TRPL) was performed to determine the abilities of charge separation and transfer in amide-COPs. As depicted in Fig. 2c, fast and consistent photocurrent responses were revealed by turning lights on and off at the same time interval. Obviously, the photocurrent intensity of amide-DAMN (2.99 μA cm^−2^) was much higher than those of amide-DAEN (1.12 μA cm^−2^) and amide-34AB (0.93 μA cm^−2^), indicating that it is the most efficient for electron–hole pair separation. After 8 cycles, the peak photocurrents did not attenuate, which can infer that the catalysts can continuously and stably output electrons and holes during visible light irradiation. EIS spectra were recorded to evaluate the ability to transport charge carriers to the reactive sites.^30^ Amide-DAMN shows the smallest radius of the semicircular Nyquist plot (Fig. S11†), reflecting the lowest charge transfer resistance. The separation and recombination of photogenerated electron–hole pairs can be measured by steady-state PL analysis, and the low intensity of the emission peak indicates that the recombination of the photogenerated electron–hole pairs is efficaciously inhibited. The order of the emission peak positions of amide-COPs is consistent with the UV-Vis absorption spectra, and amide-DAMN shows the lowest photo-luminescence intensity (Fig. S12†), indicating that it has the highest separation efficiency of carriers.^31^ As shown in Fig. 2d, amide-DAMN shows the longest average photoluminescence lifetime of 0.81 ns, which is 2.3 and 3.5 times higher than those of amide-DAEN and amide-34AB, respectively. This result means that amide-DAMN could offer more opportunities for free charges to participate in the surface photoreaction, thus exhibiting higher photocatalytic activity.

The photocatalytic CO_2_ reduction performances of amide-COPs were tested under visible-light irradiation (*λ* > 420 nm) in a CO_2_ atmosphere at room temperature. As illustrated in Fig. 2e, each amide-COP was effective for the photoreduction of CO_2_ with H_2_O, affording CO as the only carbonaceous product, and no H_2_ was detectable by GC (Fig. S13†). Amide-DAMN afforded the highest CO production rate of 20.6 μmol h^−1^ g^−1^, showing much better performance than most reported metal-free photocatalysts, and even better than many metal-containing photocatalytic systems (Table S1†). Notably, these metal-free catalysts realized the visible-light-driven photoreduction of CO_2_ at very low overpotentials that were not found in a literature survey. This may be ascribed to their unique structures.

In contrast, amide-DAMN and amide-DAEN showed different photocatalytic activities though DAMN and DAEN are *cis*–*trans* isomers. This may be attributed to the differences in the structures and band gaps of the resultant polymers, and amide-DAMN was more conducive to the photo-generated charges to reach reactive sites.

To explore the origin of CO, a control experiment was performed using amide-DAMN as the catalyst in an Ar atmosphere under visible light irradiation, and no carbonaceous product was detected. The isotope labelling experiment of ^13^CO_2_ photoreduction with H_2_O afforded ^13^CO (*m*/*z* = 29) detected by GC-MS (Fig. S14a†), indicating that CO resulted from CO_2_ reduction. Furthermore, using a mixture of H_2_^18^O (1 mL) and H_2_O (9 mL) as the reaction medium to perform the reaction, ^16^O^18^O (*m*/*z* = 34) and ^18^O_2_ (*m*/*z* = 36) were detected by GC-MS analysis, confirming that the photooxidation of H_2_O generated O_2_ (Fig. S14b†). The above results indicate that amide-COPs can realize the photocatalytic reduction of CO_2_ with H_2_O to CO and O_2_ under visible light irradiation. The fact that no H_2_ was detectable implies that the photoreduction of CO_2_ with H_2_O might follow a new mechanism different from those reported previously. In addition, amide-DAMN still retained high activity with only a slight decrease after being reused five times (Fig. S15†). TEM observation and FT-IR analysis (Fig. S16†) of the amide-DAMN sample reused 5 times indicated that no obvious morphology and structure changes were observed, unveiling its good robustness and durability in the reaction process.

To reveal the reaction mechanism for CO_2_ photoreduction with H_2_O over amide-DAMN, *in situ* FT-IR spectra were recorded after the photocatalyst was exposed to CO_2_ and water vapor for 30 min and subsequently irradiated with visible light. As shown in Fig. 2f, new peaks appeared and their intensity changed with the extension of irradiation time from 5 to 40 min. Obviously, the CO_2_ and H_2_O co-adsorbed on amide-DAMN gave rise to the signals of bidentate carbonate (b-CO_3_^2−^ at 1580 and 1358 cm^−1^),^32^ monodentate carbonate (m-CO_3_^2−^ at 1510 and 1380 cm^−1^)^33,34^ and physically adsorbed H_2_O (wide band at 1632 cm^−1^).^35^ The bands at 1684 and 1253 cm^−1^ are attributed to the vibration frequency of CO_2_^−^.^32^ It can be rationally inferred that the cyano groups and amide groups could capture CO_2_ and H_2_O and activate them. Importantly, new peaks at 1526 and 1214 cm^−1^ gradually strengthened with the prolonged irradiation time and match well with the COOH* species, giving information on the key intermediate for CO_2_ reduction to CO.^33,34^

**Fig. 3:**
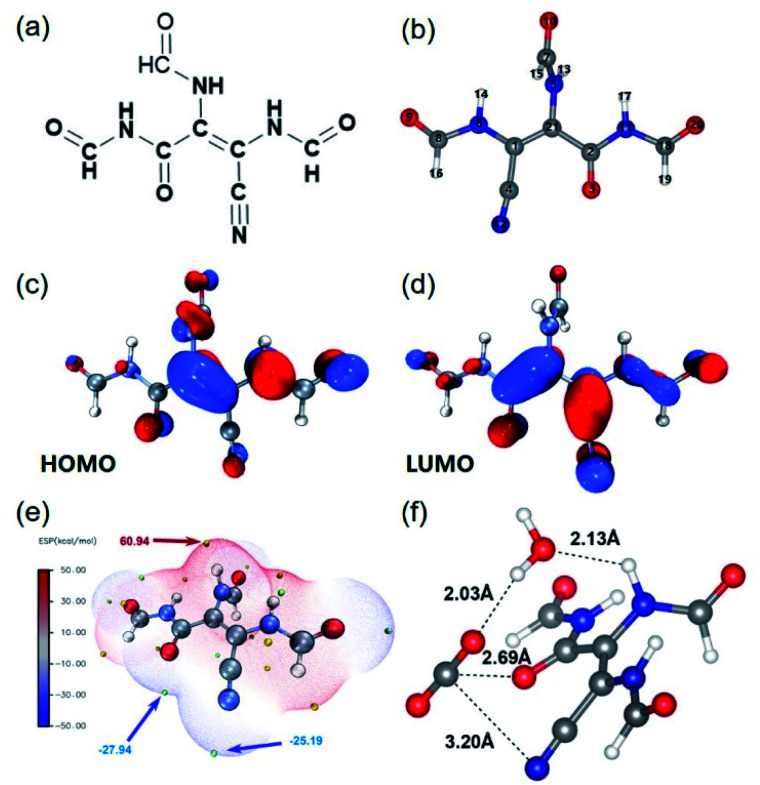
(a) Chemical structure of S-1. (b) Optimized structure of S-1, obtained by the NBO method. Blue, red, gray, and white spheres denote the positions of nitrogen, oxygen, carbon, and hydrogen atoms, respectively. (c) HOMO of S-1. (d) LUMO of S-1. (e) Electrostatic potential mapped of S-1. Color coding for ESP is from blue (negative) to red (positive). (f) The optimized structure of the intermediate in photocatalytic reactions.

To explore the photo-induced electron and charge carrier transfer in the catalyst, DFT calculations were performed taking the typical structural unit of amide-DAMN, named S-1 as shown in Fig. 3a and b, as a model compound. The natural bond orbital (NBO) analysis of the highest occupied molecular orbital (HOMO) (Fig. 3c) and the lowest unoccupied molecular orbital (LUMO) (Fig. 3d) were used to reveal the transformation of the photoexcited charge carrier in S-1. It was indicated that the HOMO of S-1 is mainly composed of the 2p_*z*_ orbitals of 1(C), 6(N) and 21(C) centers (Table S2†). These atoms can serve as light-absorption sites and produce holes to realize the oxidation of water. The LUMO of S-1 mainly consists of the 2p_*z*_ orbitals of 2(C), 3(O) and 12(N) centers (Table S3†), indicating that the photogenerated electrons could accumulate at the carbonyl/cyano groups for CO_2_ reduction. Consistent with the above results, the secondary amine group and the carbonyl/cyano groups are at the extreme points of the highest and lowest electrostatic potentials (ESPs), respectively, which are considered as potential redox active sites (Fig. 3e).

To further determine the reaction pathway, the interaction sites to attract CO_2_ on S-1 were explored by DFT calculations. As shown in Fig. S17,† both –C≡N and –C=O groups in different positions could adsorb CO_2_. Both –C≡N and –C=O groups that are adjacent to C=C have higher binding energies than –C=O that is far from C=C, indicating that the –C≡N and –C=O groups along the side of C=C have more electron cloud density due to the inductive effect (Fig. S17a–c†). In addition, it is possible for both –C≡N and –C=O to co-adsorb CO_2_ in a way as shown in Fig. S17d.† It can be inferred that CO_2_ is adsorbed and activated by carbonyl/cyano groups, at which it accepts photogenerated electrons (e^−^) from the LUMO of the catalyst to form CO_2_^−^. Meanwhile H_2_O is adsorbed and activated by the secondary amine group, at which it accepts photogenerated holes (h^+^) to realize oxidation, generating protons and releasing O_2_. From the optimized structure of the intermediate of CO_2_ reduction with H_2_O over S-1 (Fig. 3e), it is clear that one H_2_O molecule can be attracted by –NH– *via* forming a H-bond with a bond length of 2.13 Å, while one CO_2_ molecule can be co-captured by –C=O and C≡N groups with bond lengths of 2.69 and 3.20 Å, respectively. Importantly, the captured H_2_O can form a H-bond with the captured CO_2_ with a bond length of 2.03 Å. From these results, it can be deduced that the proton generated from H_2_O oxidation may directly transfer to CO_2_^−^ and involve in its reduction. This may explain why no H_2_ is generated in the reaction process.

Based on the above experimental results and analysis, a rational mechanism of photoreduction of CO_2_ with H_2_O over amide-DAMN is proposed (Fig. S18†). Upon visible-light irradiation, the photogenerated electrons accumulate at the cyano/carbonyl sites and transfer to the adsorbed CO_2_; meanwhile the photogenerated holes accumulate at the secondary amine sites and oxidize the adsorbed H_2_O, generating protons and releasing O_2_. Since the oxidation sites and the reduction sites match well, the proton generated from H_2_O oxidation may directly transfer to CO_2_^−^ at CB, thereby producing the COOH* species, which is further reduced to CO.

## Conclusions

In summary, amide-bridged COPs have been developed for the photoreduction of CO_2_ with H_2_O under visible-light irradiation without sacrificial agents and additives, which can afford CO as the sole carbonaceous product without H_2_ generation. Especially, amide-DAMN shows the highest CO production rate of 20.6 μmol g^−1^ h^−1^, much better than most reported metal-free catalysts. DFT calculations suggest that the reaction sites for CO_2_ reduction match perfectly with the sites for H_2_O oxidation, which makes the CO_2_ reduction couple well with the H_2_O oxidation, inhibiting the generation of H_2_. This work presents a new insight into designing COP photocatalysts for artificial photosynthesis.

## Experimental

### Chemicals

All reagents and solvents were purchased from commercial sources and were used without further purification, unless indicated otherwise. Diaminomaleonitrile (≥98.5%, DAMN), 2,3-diaminobut-2-ene-1,4-dinitrile (≥95%, DAEN) and dimethyl sulfoxide (≥99.9%, DMSO) was purchased from J&K China Chemical Ltd. 3,4-Diaminobenzonitrile (≥97%, 34AB) and phosphonitrilic chloride trimer (≥98%, PNC) were purchased from Innochem China Chemical Ltd.

### Catalyst preparation

Typically, 1 mmol of phosphonitrilic chloride trimer (PNC) and 6 mmol of diaminomaleonitrile (DAMN) were, respectively, dissolved in 10 mL of ultra-dry DMSO. At 140 °C, under an argon atmosphere, the DMSO solution of DAMN was added dropwise into the DMSO solution of PNC. The mixture was stirred for 18 h at 140 °C. After being cooled to room temperature, 20 mL of water was added to the reaction system, and then the solid was collected *via* filtration, followed by washing with distilled water, tetrahydrofuran and ethanol several times. After being extracted using a Soxhlet extractor with ethanol, H_2_O and THF (1 : 1 : 1) for 48 h, the final sample was obtained after being dried at 80 °C in a vacuum for 12 h, and named amide-DAMN.

Similarly, using 2,3-diaminobut-2-ene-1,4-dinitrile (DAEN) and 3,4-diaminobenzonitrile (34AB) as the monomers, the corresponding polymers, named amide-DAEN and amide-34AB, were obtained.

### Photocatalytic carbon dioxide reduction experiment

In a typical photocatalytic experiment, 5 mg of polymer catalyst dispersed in 1 mL of ethanol was immobilized onto a glass sheet of 3 cm × 3 cm, and ethanol was then evaporated using an infrared lamp. The glass sheet with the catalyst was moved into a 250 mL photoreactor with 10 mL of H_2_O and suspended on the top of the reactor. Prior to illumination, the reactor was vacuumed and was subsequently backfilled with ultra-pure CO_2_ (99.999%) for about 1 h to reach the adsorption/desorption equilibrium of CO_2_ on the surface of the polymer catalyst. The pressure of the reactor was 1 atm and the temperature was kept at 25 °C using circulating water. Then the reactor was illuminated for a desired time (*e.g.*, 10 h) with a 300 W Xe lamp (Aulight CEL-HX, Beijing) equipped with a 420 nm cut-off filter positioned 5 cm above the reactor. After irradiating for 10 h, 2 mL of the gaseous mixture was periodically sampled from the glass reactor at a given time interval, and analyzed by using a gas chromatograph equipped with a thermal conductivity detector (4890, Agilent Technology) with Ar as the carrier gas. The isotope-labelling experiment was performed using ^13^CO_2_ instead of ^12^CO_2_, H_2_^18^O (1 mL) and H_2_^16^O (9 mL) and the gaseous products were analyzed using gas chromatography-mass spectrometry.

## Data availability

All characterization, computational methods and data, copies of FT-IR, CP/MAS ^13^C NMR, XPS, XRD, EIS, photoluminescence spectra, TEM/SEM images, N_2_ sorption isotherms and pore size distribution, CO_2_ adsorption capacity isotherms, Tauc plots together with the bandgaps, and Motto–Schottky plots of the photocatalysts, GC spectrum of CO_2_ photoreduction products, GC-MS spectrum of ^13^CO_2_ photoreduction, calculation data, related to this study, can be found in the ESI.†

## Author contributions

The manuscript was written through contributions of all authors. All authors have given approval to the final version of the manuscript.

## Conflicts of interest

There are no conflicts to declare.

## Supplementary Material

SC-012-D1SC02499J-s001
