# Supplementary material for: Amide-bridged conjugated organic polymers: efficient metal-free catalysts for visible-light-driven CO2 reduction with H2O to CO
Source: Chem Sci. 2021 Jul 13;12(34):11548–53. doi: 10.1039/d1sc02499j (PMC8447930; doi:10.1039/d1sc02499j)
Supplement: SC-012-D1SC02499J-s001 [file SC-012-D1SC02499J-s001.pdf]

Electronic Supplementary Material (ESI) for Chemical Science.
This journal is © The Royal Society of Chemistry 2021

Supporting Information for

Amide-Bridged Conjugated Organic Polymers: Efficient Metal-Free Catalysts for Visible-Light-Driven CO_2 Reduction with H_2O to CO

Fan Wen,‡[ac] Fengtao Zhang,‡[ab] Zhen Wang,[ab] Xiaoxiao Yu,[ab] Guipeng Ji,[a] Dongyang Li,[ac] Shengrui Tong,[a] Yingbin Wang,[c] Buxing Han,[ab] Zhimin Liu*[ab]

a Beijing National Laboratory for Molecular Sciences, Key Laboratory of Colloid and Interface and Thermodynamics, CAS, Research/Education Center for Excellence in Molecular Sciences, Institute of Chemistry, Chinese Academy of Sciences, 100190 Beijing (P. R. China). E-mail: liuzm@iccas.ac.cn.

b School of Chemistry, University of Chinese Academy of Sciences, Beijing 100049 (P. R. China)

c School of Science, China University of Geosciences, Beijing, 100083 (P. R. China)

‡ These authors contributed equally

Characterization

FTIR spectra of the samples were collected on a TENSOR 27 FTIR at a resolution of 2 cm^{-1}. Solid-state NMR experiments were performed on a Bruker WB Avance II 400 MHz spectrometer. The ^{13}C CP/MAS NMR spectra were recorded with a 4 mm double-resonance MAS probe and with a sample spinning rate of 10.0 kHz; a contact time of 2 ms (ramp 100) and pulse delay of 3 s were applied. X-ray photoelectron spectroscopy (XPS) was performed on an ESCAL Lab 220i-XL spectrometer at a pressure of ~3×10^{-9} mbar (1 mbar = 100 Pa) using Al K_{α} as the excitation source (1486.6 eV) and operated at 15 kV and 20 mA. The binding energies were referenced to the C 1s line at 284.8 eV from adventitious carbon. Field emission scanning electron microscopy (SEM) observations were performed on a Hitachi S-4800 microscope operated at an accelerating voltage of 15.0 kV. Transmission electron microscopy (TEM) images were obtained with a JEOL JEM-1011 and Hitachi HT7700 instrument operated at 200 kV. Gas sorption isotherms were obtained with Micromeritics TriStar II 3020 and Micromeritics ASAP 2020 M+C accelerated surface area and porosimetry analyzers at certain temperature. The samples were outgassed at 150 ^{o}C for 8 h before the measurements. Surface areas were calculated from the adsorption data using Brunauer-Emmett-Teller (BET) methods. The pore-size-distribution curves were obtained from the adsorption branches using Barrett-Joyner-Halenda (BJH) method. The XRD analysis was performed on a D/MAX-RC diffractometer operating at 30 kV and 100 mA with Cu K_{α} radiation. The UV–vis diffused reflectance spectra (UV-DRS) were collected on UV-2600 using $BaSO_4$ as a reference. PL emission spectra were obtained on FluoroMax+ fluorescence spectrophotometer with 370 nm excitation (HORIBA, United States). The time-resolved fluorescent decay spectroscopy was performed on FLS980 with 405 nm excitation (Edinburgh Instruments, UK). The reaction mixture was analyzed by means of GC (Agilent 7890B) equipped with a TCD detector and a packed column (carbon molecular sieve TDX-01, 3 mm in diameter and 1 m in length) using argon as the carrier gas. The column oven was temperature-programmed with a 2 min initial hold at 333 K, followed by the temperature increase to 473 K at a rate of 20 K/min and kept at 273 K for 21 min. The in situ FTIR spectra were recorded in the range from 4000 to 650 cm^{-1} with a Nicolet FTIR Spectrometer 6700 equipped with a liquid-nitrogen-cooled narrow band mercury-cadmium-telluride (MCT) detector.

Photoelectrochemical characterization

Electrochemical measurements were conducted with a CorrTest electrochemical station in a conventional three-electrode cell, using a Pt plate as the counter electrode and a 3 mol/L Ag/AgCl as the reference electrode. The working electrode was prepared by spreading aqueous slurry of photocatalyst (50 µL) over ITO glass substrate, and the coated area was fixed at 1 cm^2. The suspension was prepared by dispersing photocatalyst (50 mg) with Nafion solution in ethanol (1 wt %, 2 mL). The working electrode was irradiated from its back. The supporting electrolyte was an aqueous solution of Na_2SO_4 (0.2 M), which was purged with nitrogen for 1.5 h before measurement.

Computational methods

All DFT calculations in this study were performed using Gaussian 09 package.[1] The M06-2X functional with def-tzvp basis set was employed, coupled with Grimme's D3 dispersion correction to perform geometry optimization.[2] All calculated structures were verified with no imaginary frequency (IF). Besides, an ultrafine integration grid (99,590) was used for numerical integrations. Thermal corrections were carried out with harmonic frequency analysis using Shermo code on optimized structures under T=298.15 K and 1 atm pressure.[3] The binding energies were evaluated at M06-2X functional with def2tzvp basis set according to the previous optimized structures. NAO were carried out by Natural bond orbital (NBO) analysis to obtain the composition of atomic orbitals of each atom in HOMO and LUMO.[4] The VESTA molecular visualizing program was employed to draw 3D molecular structures,[5] the VMD [6] and Multiwfn[7] program was employed to perform analysis of electrostatic potential (ESP) energy and draw picture.

Results of Characterizations

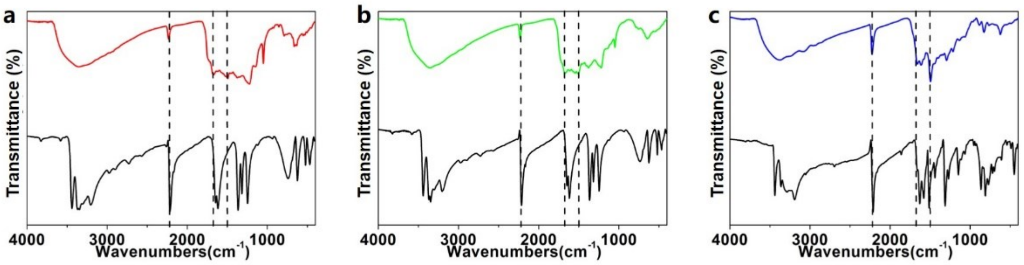

Fig. S1 FT-IR spectra of (a) DAMN and Amide-DAMN, (b) DAEN and Amide-DAEN, (c) 34AB and Amide-34AB.

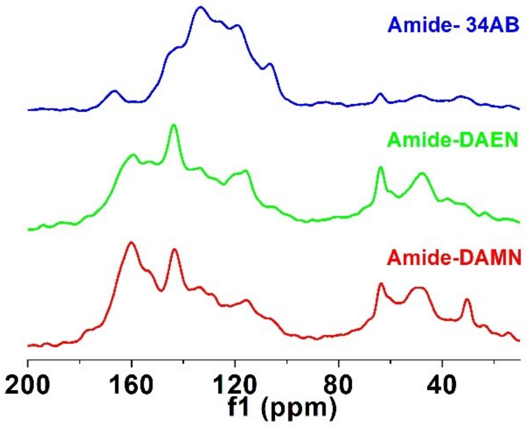

Fig. S2 (CP/MAS) ^{13}C NMR spectra of Amide-COPs.

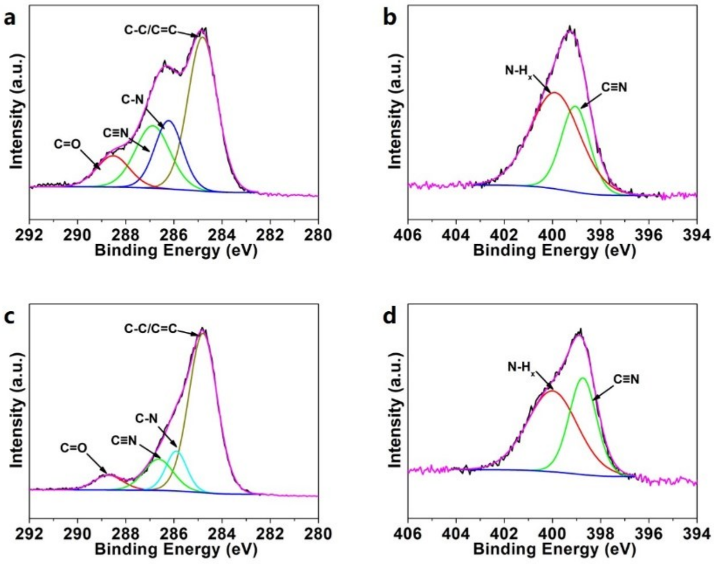

Fig. S3 XPS spectra of (a) C 1s and (b) N 1s for Amide-DAEN. XPS spectra of (c) C 1s and (d) N 1s for Amide-34AB.

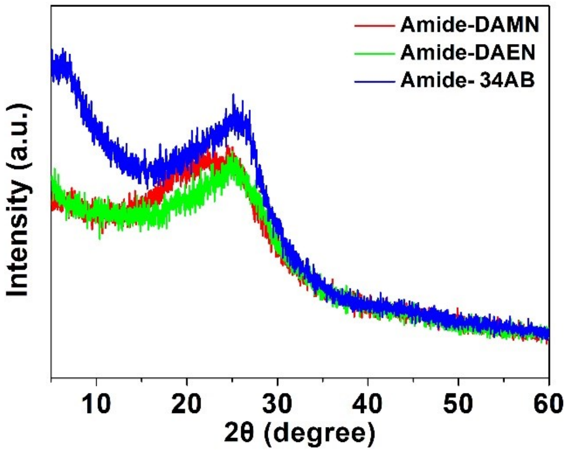

Fig. S4 Powder X-ray diffraction spectra of Amide-COPs.

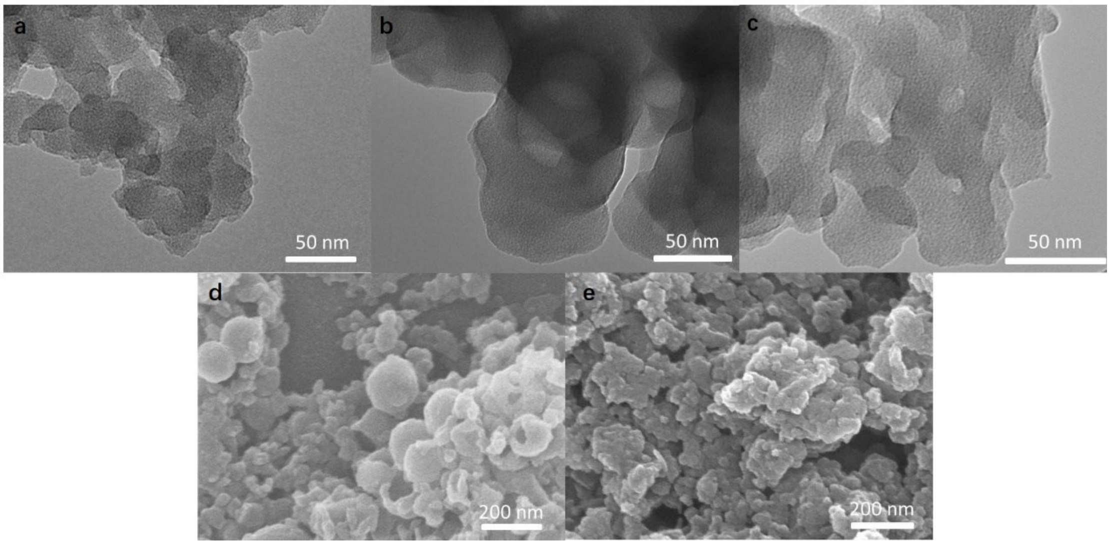

Fig. S5 (a) TEM of Amide-DAMN. (b) TEM and (d) SEM of Amide-DAEN. (c) TEM and (e) SEM of Amide-34AB.

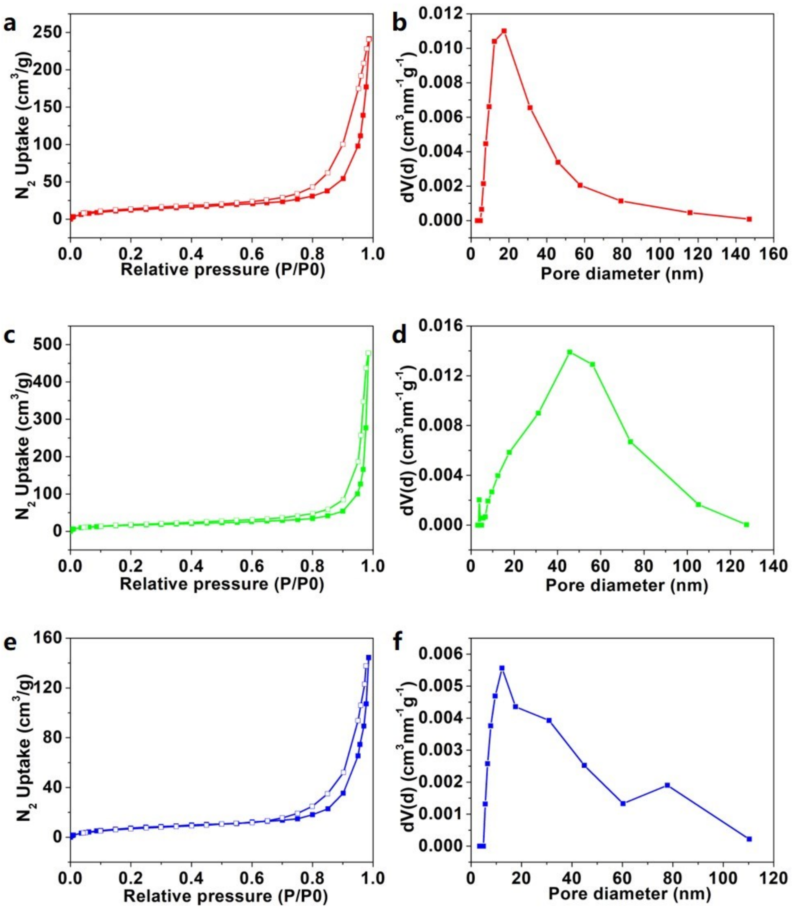

Fig. S6 (a) Adsorption (filled) and desorption (empty) isotherms of N_2 at 77 k for Amide-DAMN. (b) BJH pore size distribution of Amide-DAMN. (c) Adsorption (filled) and desorption (empty) isotherms of N_2 at 77 k for Amide-DAEN. (d) BJH pore size distribution of Amide-DAEN. (e) Adsorption (filled) and desorption (empty) isotherms of N_2 at 77 k for Amide-34AB. (f) BJH pore size distribution of Amide-34AB.

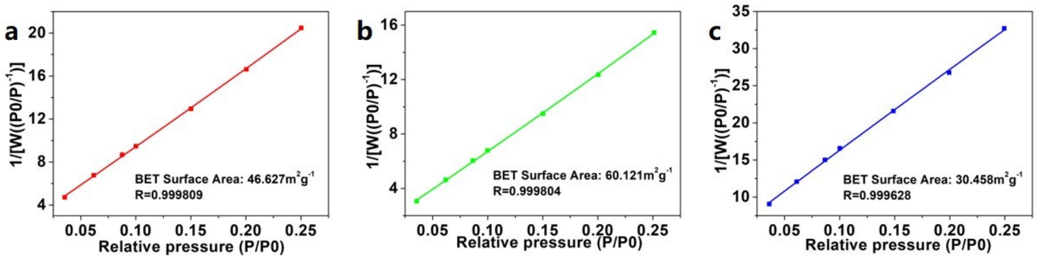

Fig. S7 BET plot of (a) Amide-DAMN, (b) Amide-DAEN and (c) Amide-34AB.

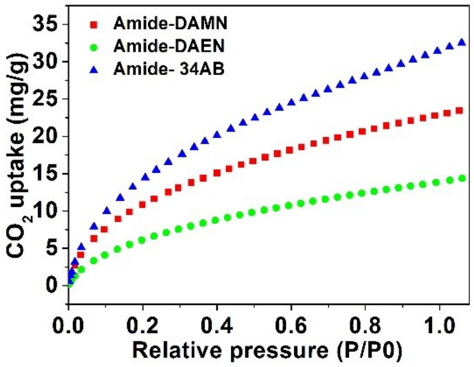

Fig. S8 CO_2 adsorption capacity isotherms of Amide-COPs at 273 K.

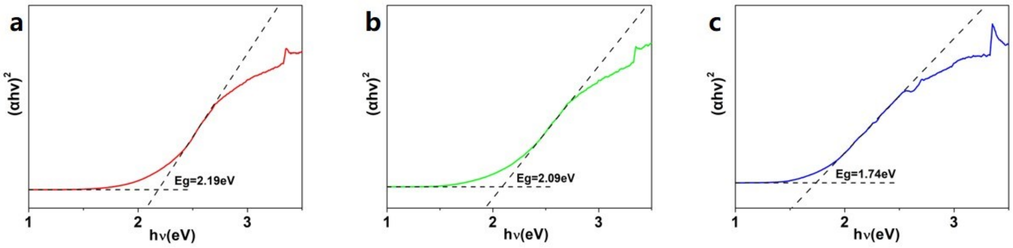

Fig. S9 Tauc plots together with the bandgaps for: (a) Amide-DAMN, (b) Amide-DAEN and (c) Amide-34AB.

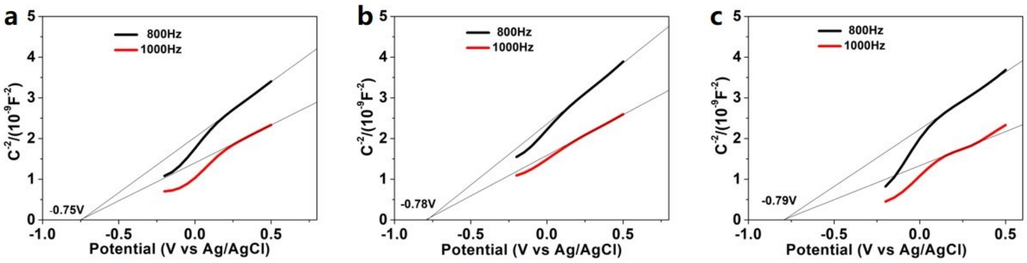

Fig. S10 Motto-Schottky plots of Amide-COPs. Electrode in 0.2 M Na_2SO_4 (pH=6.8). (a) Amide-DAMN, (b) Amide-DAEN and (c) Amide-34AB.

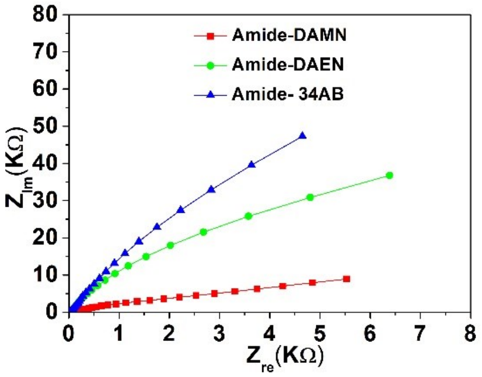

Fig. S11 EIS spectra of Amide-COPs at 0.01 V bias potential vs Ag/AgCl in 0.2 M Na_2SO_4 (pH=6.8).

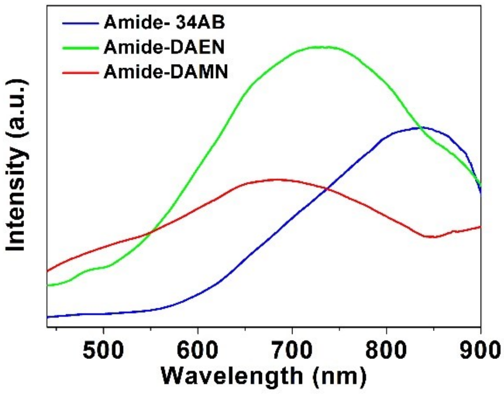

Fig. S12 Photoluminescence spectra of Amide-COPs at room temperature.

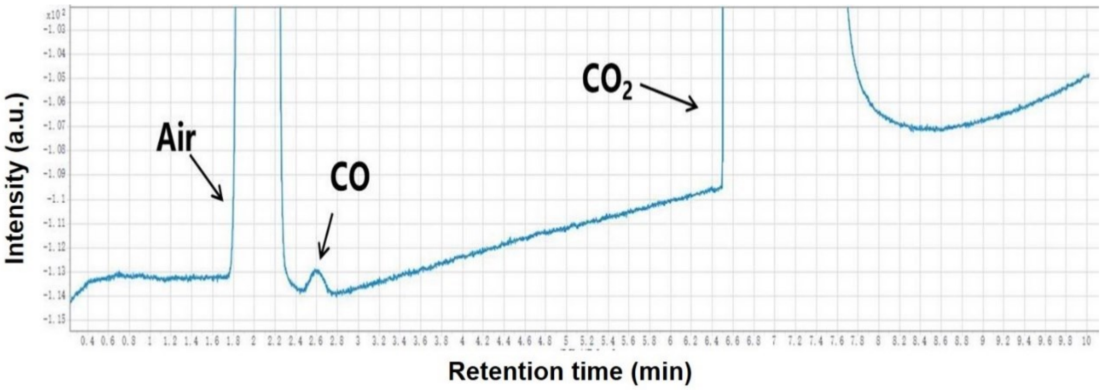

Fig. S13 Typical GC spectrum of CO_2 photoreduction products over Amide-DAMN. Retention time: 0.78 min (H_2), 1.76 min (Air), 2.48 min (CO), 4.55 min (CH_4) and 6.51 min (CO_2).

Table S1. Comparison with other photocatalysts and photocatalytic systems employed for CO_2 reduction under visible light irradiation.

Photocatalyst	Reaction conditions	Main reaction products (μmol/g/h)	Main reaction selectivity /%	Reference
NOP-COP	300 W Xe lamp (λ>420 nm) TEOA	CH4: 22.5	90.2	8
PEosinY-1	300 W Xe lamp (λ>420 nm)	CO: 33	92	9
TTCOF-Zn	300 W Xe lamp (800 nm>λ>420 nm)	CO: 2.06	100	10
CdS/N-doped graphene	350 W Xe lamp (λ>420 nm)	CO: 2.59	88.7	11
HCP-TiO_2-FG	300 W Xe lamp (λ>420 nm)	CH4: 27.62	56.1	12
Amide-DAMN	300 W Xe lamp (λ>420 nm)	CO: 20.6	100	*This work.*
Amide-DAEN	300 W Xe lamp (λ>420 nm)	CO: 16.3	100	*This work.*

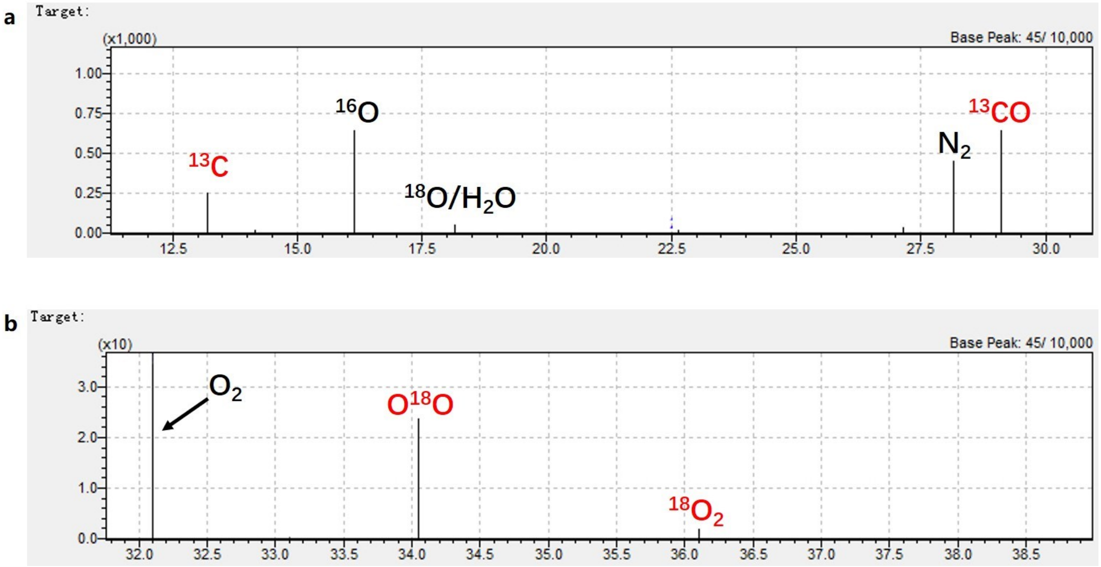

Fig. S14 (a) GC-MS spectrum of ^{13}CO generated from the photocatalytic reaction of $^{13}CO_2$ over Amide-DAMN under visible light irradiation. (b) GC-MS spectrum of $O^{18}O$ and $^{18}O_2$ generated from the photocatalytic reaction of $H_2{}^{18}O$ over Amide-DAMN under visible light irradiation.

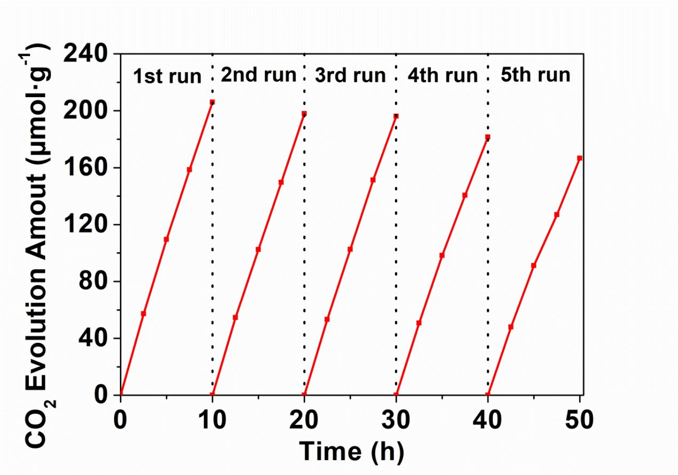

Fig. S15 Recyclability test of Amide-DAMN under visible light irradiation.

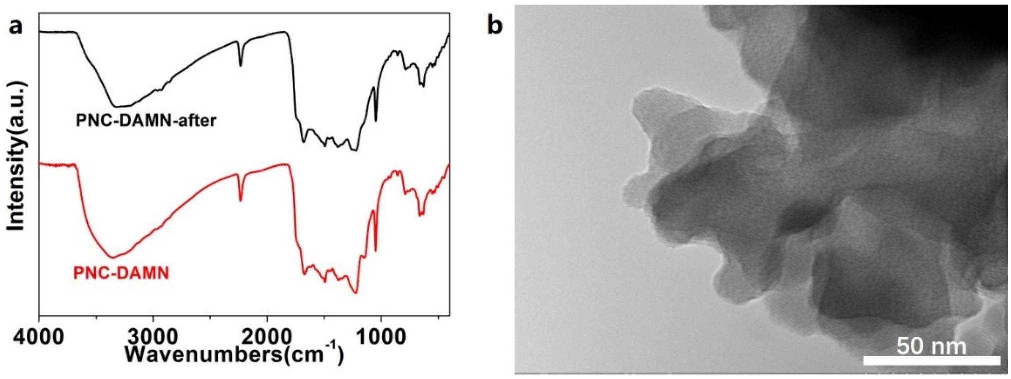

Fig. S16 (a) FT-IR spectra of Amide-DAMN and Amide-DAMN-after recorded as KBr pellets. (b) TEM image of Amide-DAMN sample after photocatalytic reaction.

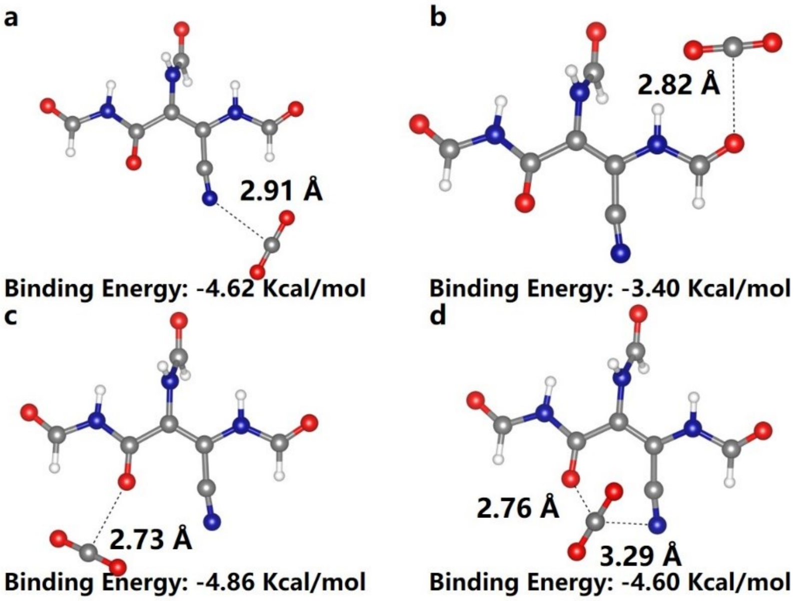

Fig. S17 The binding distance and binding energy between cyano or carbonyl groups in S-1 and CO_2.

Table S2. The details of the HOMO composition of S-1 and orbital compositions of the atoms.

Center	Composition/%	Center	Composition/%	Center	Composition/%
1(C)	9.191	8(C)	0.142	15(H)	0.429
2(C)	0.257	9(O)	9.694	16(H)	0
3(O)	5.570	10(O)	3.376	17(H)	0
4(C)	0.331	11(N)	1.725	18(C)	0.016
5(N)	4.888	12(N)	3.473	19(H)	0.020
6(N)	23.823	13(H)	1.259	20(O)	1.102
7(C)	0.632	14(H)	0.016	21(C)	33.137

Table S3. The details of the LUMO composition of S-1 and orbital compositions of the atoms.

Center	Composition/%	Center	Composition/%	Center	Composition/%
1(C)	26.641	8(C)	4.309	15(H)	0.049
2(C)	9.861	9(O)	4.221	16(H)	0
3(O)	7.958	10(O)	0.155	17(H)	0
4(C)	3.376	11(N)	1.465	18(C)	2.614
5(N)	0.141	12(N)	11.850	19(H)	0
6(N)	3.358	13(H)	0.753	20(O)	2.569
7(C)	0.611	14(H)	0.002	21(C)	16.864

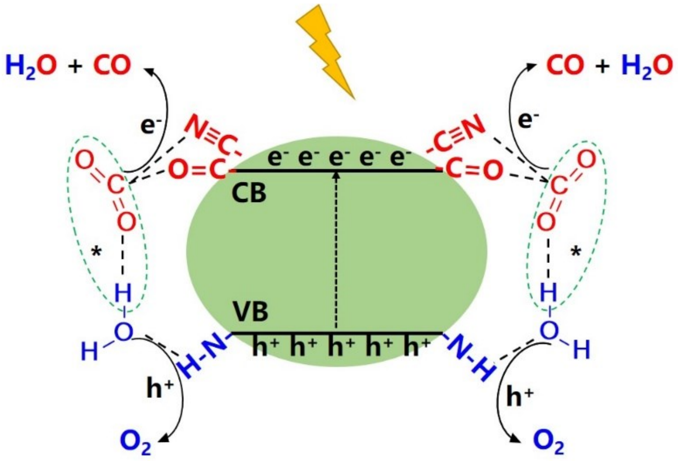

Fig. S18 Schematic of the mechanism of Amide-COPs CO_2RR with H_2O oxidation.

References

[1] Gaussian 09, Revision D.01, M. J. Frisch, G. W. Trucks, H. B. Schlegel, G. E. Scuseria, M. A. Robb, J. R. Cheeseman, G. Scalmani, V. Barone, G. A. Petersson, H. Nakatsuji, X. Li, M. Caricato, A. Marenich, J. Bloino, B. G. Janesko, R. Gomperts, B. Mennucci, H. P. Hratchian, J. V. Ortiz, A. F. Izmaylov, J. L. Sonnenberg, D. Williams-Young, F. Ding, F. Lipparini, F. Egidi, J. Goings, B. Peng, A. Petrone, T. Henderson, D. Ranasinghe, V. G. Zakrzewski, J. Gao, N. Rega, G. Zheng, W. Liang, M. Hada, M. Ehara, K. Toyota, R. Fukuda, J. Hasegawa, M. Ishida, T. Nakajima, Y. Honda, O. Kitao, H. Nakai, T. Vreven, K. Throssell, J. A. Montgomery, Jr., J. E. Peralta, F. Ogliaro, M. Bearpark, J. J. Heyd, E. Brothers, K. N. Kudin, V. N. Staroverov, T. Keith, R. Kobayashi, J. Normand, K. Raghavachari, A. Rendell, J. C. Burant, S. S. Iyengar, J. Tomasi, M. Cossi, J. M. Millam, M. Klene, C. Adamo, R. Cammi, J. W. Ochterski, R. L. Martin, K. Morokuma, O. Farkas, J. B. Foresman, and D. J. Fox, Gaussian, Inc., Wallingford CT, 2016.

[2] E. Caldeweyher, C. Bannwarth, S. Grimme, *The Journal of Chemical Physics,* 2017, **147**, 034112

[3] T. Lu, Q. Chen, *ChemRxiv,* 2020, DOI: 10.26434/chemrxiv.12278801.v1

[4] E. D. lendening, J. K. Badenhoop, A. E. Reed, J. E. Carpenter, J. A. Bohmann, C. M. Morales F. Weinhold, Theoretical Chemistry Institute: University of Wisconsin, Madison, 2001.

[5] K. Momma, F. Izumi, *J. Appl. Crystallogr*., 2011, **44**, 1272–1276.

[6] Y. Yamada, S. Gohda, K. Abe, T. Togo, N. Shimano, T. Sasaki, H. Tanaka, H. Ono, T. Ohba, S. Kubo, T. Ohkubo, S. Sato, *Carbon N. Y.,* 2017, **122**, 694–701.

[7] T. Lu, F. Chen, *J. Comput. Chem.,* 2012, **33**, 580–592.

[8] S. Guo, H. Zhang, Y. Chen, Z. Liu, B. Yu, Y. Zhao, Z. Yang, B. Han, Z. Liu, *ACS Catal.,* 2018, **8**, 4576–4581.

[9] X. Yu, Z. Yang, B. Qiu, S. Guo, P. Yang, B. Yu, H. Zhang, Y. Zhao, X. Yang, B. Han, Z. Liu, *Angew. Chem., Int. Ed.,* 2019, **58**, 632–636.

[10] M. Lu, J. Liu, Q. Li, M. Zhang, M. Liu, J.-L. Wang, D.-Q. Yuan, Y.-Q. Lan, *Angew. Chem., Int. Ed*., 2019, **58**, 12392–12397.

[11] C. Bie, B. Zhu, F. Xu, L. Zhang, J. Yu, *Adv. Mater.,* 2019, **31**,1902868.

[12] S. Wang, M. Xu, T. Peng, C. Zhang, T. Li, I. Hussain, J. Wang, B. Tan, *Nat. Commun.,* 2019, **10**, 676.
